# Peer mentoring to support learning in the life sciences

**DOI:** 10.1042/ETLS20253022

**Published:** 2026-02-23

**Authors:** Beatrice Hayes, Nura Sidarus, Narender Ramnani, James P. McEvoy, Nicholas S. Freestone

**Affiliations:** 1 School of Life Sciences and the Environment, Royal Holloway University of London, Egham, Surrey, TW20 0EX, U.K.; 2 Department of Pharmacy, Kingston University, 55-59 Penrhyn Road, Kingston upon Thames, London, KT1 2EE, U.K.

**Keywords:** awarding gaps, belonging, education, inclusive education, peer mentoring

## Abstract

We present a pair of case studies of undergraduate peer mentoring schemes in schools of life science to support student transition and reduce demographic awarding gaps. The first, PEMENTOS (PEer MEntoring TO Succeed), was piloted at Royal Holloway University of London to ease the transition to university for students from Black and Global Majority (BGM) backgrounds by fostering belonging and informal knowledge-sharing. The second, Kingston University’s Academic Peer Mentoring Programme, was an institution-wide initiative offering flexible, department-led models of academic and psychosocial support. Evaluation of PEMENTOS showed increased mentee confidence, engagement and academic performance, with narrowing first-year grade gaps between BGM and White students. Mentees at Kingston, as shown by an analysis of their results across the university and in the Pharmaceutical Science course in particular, demonstrated greater pass and progression rates, with large gains among BTEC-qualified and commuting students. Both programmes also yielded developmental benefits for mentors. These case studies suggest that peer mentoring programmes can enhance undergraduate student success and inclusion in the life sciences. Key shared features – a high level of programme structure, alongside mentor training and compensation – may be particularly important.

## Introduction

Peer mentoring schemes have been widely employed in higher education, but variations in programme design and evaluation make it hard to identify the elements most likely to be effective in the life sciences. We therefore present a pair of peer mentoring case studies drawn from schools of life science in two universities with demographically diverse student bodies, as detailed below. The schemes had several effective elements in common, along with differences of approach and scale. In both schemes, first-year undergraduates were mentored by more experienced students in a structured sequence of in-person meetings, and mentors were trained and paid for their work. Both programmes aimed to improve the assessment results of certain groups of students but were open to all. The Royal Holloway scheme, PEMENTOS, was a pilot organised within the School of Life Sciences and the Environment that aimed to reduce ethnic demographic awarding gaps by easing students’ transition to higher education. PEMENTOS mentors offered informal support, shared lived experiences and helped mentees navigate the ‘hidden curriculum’ of university life. The Kingston University intervention, a more mature programme that has been running since 2016, aimed to improve progression rates through an academic peer mentoring scheme flexibly organised across the whole university. In light of the significant educational disruption experienced by students entering higher education post-2020, the scheme was positioned as a critical intervention to mitigate the long-term impacts of disrupted prior learning. Academic Peer Mentors at Kingston University provided structured academic assistance and guided mentees through challenging topics. While the programme shared some similarities with the well-established Peer Assisted Learning (PAL) framework [1], it also incorporated significant distinctions. Results from both programmes demonstrate success, suggesting the effectiveness of the schemes’ shared design elements.

## PEMENTOS: a transition-focused peer mentoring scheme

### Background and demographics

PEMENTOS (PEer MEntoring To Succeed) was run within the School of Life Sciences and the Environment at Royal Holloway University of London in the 2022/23 academic year. It was co-ordinated as an EDI project by the school’s EDI Committee and its collaborators. The school comprised five departments (Biological Sciences, Earth Sciences, Geography, Health Studies and Psychology) and 1680 full-time equivalent undergraduate students, of whom 567 were in Year 1 (Y1, Level 4). The school had seen a recent rise in the ethnic diversity of its students, with BGM numbers roughly doubling from 2017 to 2023. By the end of this period, 43% of students were from Black and Global Majority (BGM) communities: 25% were Asian, 8% Mixed Ethnicity and 5% were Black. (For comparison, around 18% of the population of England and Wales and 16% of the local population are BGM [2,3].) Rapid ethnic diversification was accompanied by smaller changes in socioeconomic status. From 2020 to 2023, the proportion of the school’s undergraduates coming from the most deprived backgrounds (quintiles 1 and 2 in the Index of Multiple Deprivation [IMD]) rose from 25% to 27%, and the proportion from the lowest areas of HE participation (quintiles 1 and 2 in Participation of Local Areas 4 [POLAR4]) fell slightly, from 14% to 13%.

In response to ethnic diversification, which was reflected across the university and posed specific challenges in student support, Royal Holloway trialled ethnic awarding-gap interventions at the supra-departmental level. Although pandemic-related grade inflation in the School of Life Sciences and the Environment had reduced the BGM/White ‘good degree’ (2:1 or 1st class) awarding gap [4] in the 2021/22 academic year to 3 percentage points (pp), ethnic awarding gaps in the school were persistent and differed within BGM ethnicities. In 2021/22, the mean grades of Asian students in Y1 were 6 pp lower than those of White students in the same year. The mean grades of Black students were 10 pp lower than those of White students across all years, and this difference was found to have fully emerged in Y1. Royal Holloway’s Access and Participation Plan (APP) notes that the proportion of Black students awarded a 1st class or 2:1 degree classification in 2021/22 was 68.6%, compared with 90.0% for White students: a gap of 21.4 pp across the university [5].

Given this evidence and guided by the literature, we hypothesised that the school’s ethnic awarding gaps were associated with students’ experience of a more challenging transition into higher education and a reduced sense of belonging [6]. A substantial body of research shows that belonging is strongly linked to student engagement, wellbeing, continuation and progression, and that it is often lowest among minoritised learners. Large-scale survey analysis suggests that the most important predictors of belonging are the quality of the overall educational experience and the support provided to settle into the institution [7]. Complementing this finding, a recent critical review emphasises that belonging is influenced by multiple institutional factors, including personalised support, inclusive cultures and pedagogical practices that facilitate peer connection [8].

Peer mentoring programmes have been shown to improve feelings of belonging [9] and work in a variety of ways, including coaching, role modelling, psychological support and goal setting [10]. They have also been associated with improved academic performance [11]. PEMENTOS was intended to boost the achievement of minoritised students in the School of Life Sciences and the Environment through a similar range of mechanisms. Mentors did not provide specific academic support or tutoring; rather, mentors were asked to provide informal support by sharing their own experiences. This could help reveal the ‘hidden curriculum’ and implicit knowledge of navigating university life, especially to those who are the first in the family to go to university. Mentors also provided general information and signposting to school and university services. We thus hoped to ease the transition of demographically disadvantaged undergraduates into the school, to increase their sense of belonging and to reduce ethnic awarding gaps.

### Scheme design

Following a successful internal funding application and ethical approval, the PEMENTOS organising team was formed to include a number of academic staff with inclusivity leadership responsibilities within the school, supported part time by a member of the school’s professional services team. The team invited applications from continuing undergraduates and postgraduates in the school to mentor newly arrived Year 1 students in the first 12-week term of the 2022/23 academic year. Mentors were paid for their work [12]; in the interests of fairness, the programme was open to, and advertised to, all students. Incoming Year 1 students were invited to join as mentees in the summer before they arrived at Royal Holloway. The scheme involved 68 mentors and 103 mentees in 2022/23. Mentors and mentees were predominantly women (>70% in all surveys; for comparison, 77% of the school UG population were women) with a roughly equal split between White and BGM ethnicities. Mentors and mentees were assigned to small groups in a ratio of 1:1 or 1:2, and mentees were matched, as far as possible, with mentors studying in the same department. Demographic information was collected in the pre-scheme questionnaire but not used for matching unless this was requested by the mentee.

**Figure 1:**
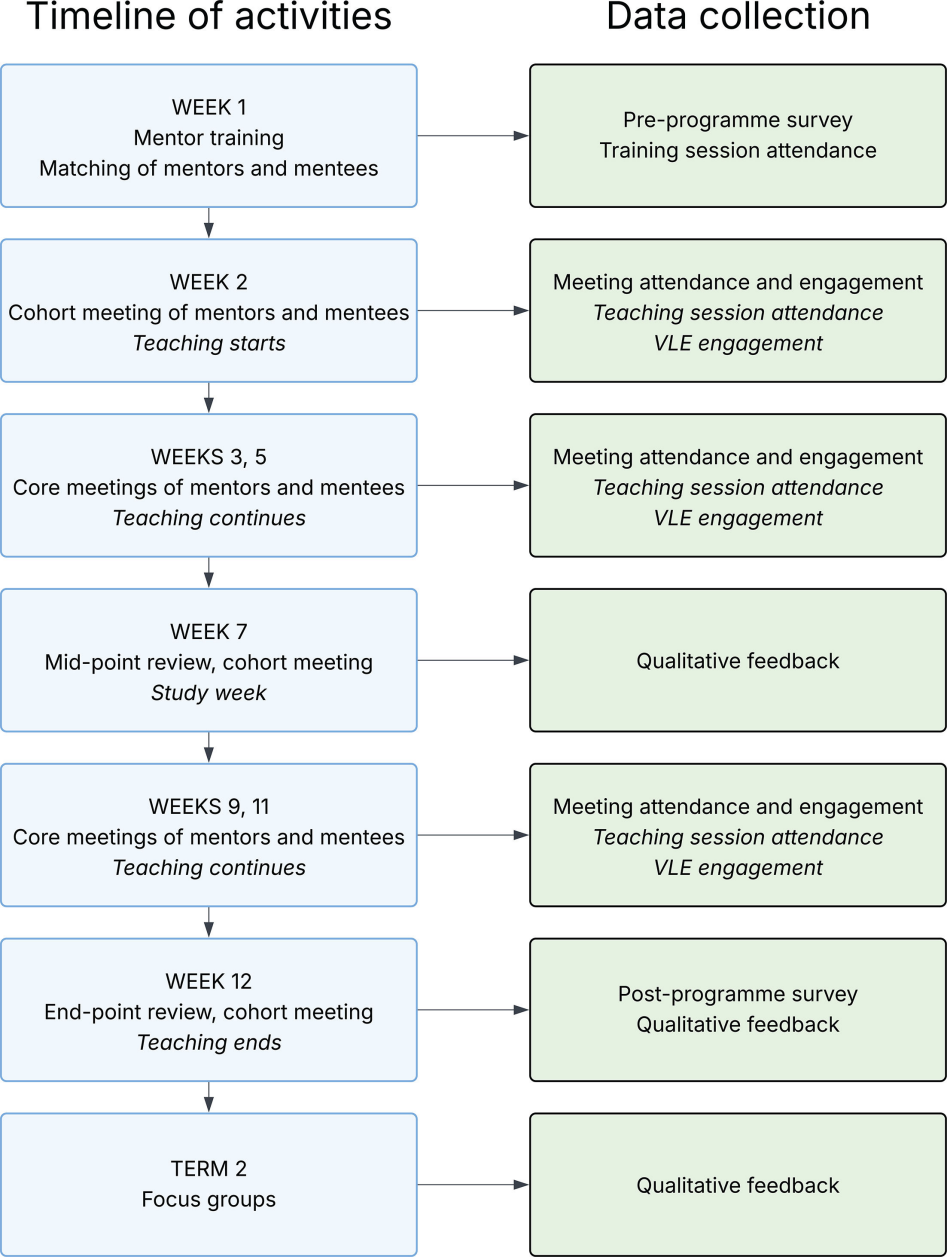
Timeline of the PEMENTOS programme and associated data collection. Week numbers refer to weeks in Term 1 of the 2022/23 academic year at Royal Holloway University of London.

PEMENTOS was designed as an intensive, structured programme and all meetings took place in person. Microsoft Teams and Forms were used to facilitate communication within the organising team and with mentors, and Qualtrics was used for pre- and post-programme questionnaires. In Week 1, mentors attended a mandatory training session that was designed and led by the PEMENTOS organising team, delivering key information and involving role-playing exercises. Mentors were also given a 15-page digital handbook which included details of the scheme’s organisation and the mentor’s role, the university’s various support systems, and ideas for conversation topics to maximise mentee engagement. This was followed by a cohort meeting of all the mentors and mentees in Week 2, the first teaching week of term. There followed a bi-weekly schedule of core mentor–mentee meetings throughout the term, with mid-term and end-of-term review meetings to build cohort identity and gather feedback. Figure 1 summarises these activities and associated data collection.

### Results and Discussion

**Figure 2:**
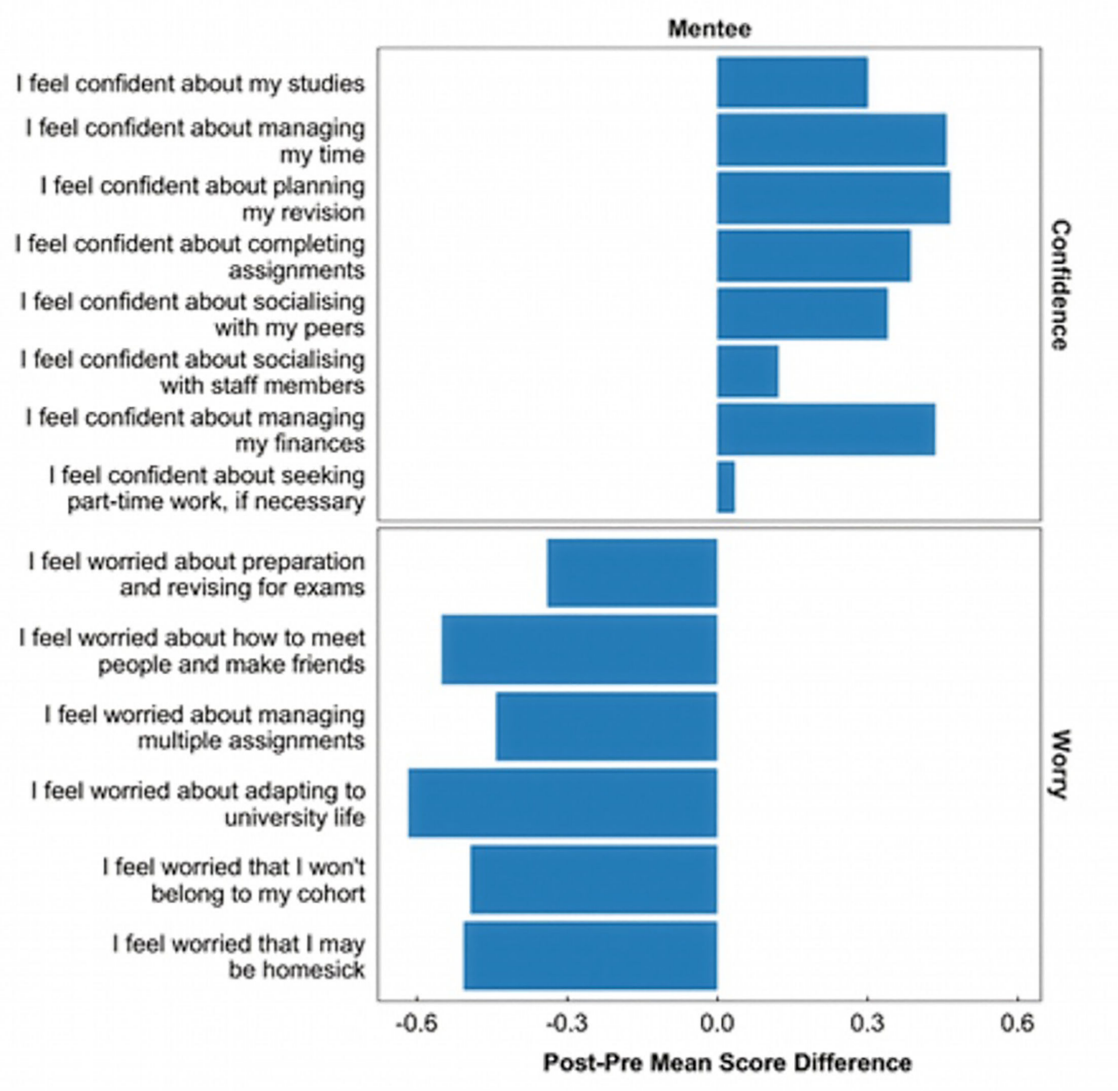
Pre-post survey results (*N* = 70, *n* = 29 respectively) for mentees’ responses to the 14 common Likert-scale questions. Ratings indicate agreement with statements on a five-point scale: 1 = strongly disagree, 2 = somewhat disagree, 3 = neither agree nor disagree, 4 = somewhat agree, 5 = strongly agree. Questions are grouped by phrasing into ‘confidence’ and ‘worry’ categories. These terms reflect the language of the PEMENTOS survey items and represent students’ self-reported perceptions, not standardised psychological constructs.

Figure 2 shows that surveyed mentees were significantly more confident and less worried about university life by the end of the PEMENTOS programme.

The biggest increases in confidence were seen in response to the statements on feeling confident about ‘managing my time’ and ‘planning my revision’, and the biggest reductions in worry were seen in response to statements on feeling worried about ‘adapting to university life’ and ‘how to meet people and make friends’. BGM mentees reported a greater increase in confidence about socialising with their peers and about managing their finances than White mentees did. Both mentees and mentors gave predominantly favourable qualitative feedback: one mentee commented that ‘I liked… being able to talk to someone who has gone through the same thing as you. (My mentor) provided good advice and ensured not only my studies were going well but also regularly asking about wellbeing’. Mentees saw value in peer support, reporting an increased sense of belonging and appreciating mentors as an accessible source of practical advice. Mentors reported gains in transferable skills and valued the opportunity to reflect on their own personal growth during their time at university. Mentors’ stated motivations for their work were both strongly altruistic and financially pragmatic, confirming the importance of paying students for such work. The challenges they reported included confidentiality concerns, difficulties in scheduling meetings and occasional mentee disengagement.

Turning to engagement with their studies, mentees logged into the virtual learning environment (VLE) at almost twice the rate of non-participants, and the rate of VLE assessment submissions was higher for mentees than for non-participants. Attendance of mentees at teaching sessions was also higher than that of non-participants.

Across all of the Y1 modules in the School in 2022/23, the average grades of mentees were about 4 pp higher than each of the module averages. The average Y1 grade gap between Black and White students decreased from 10 pp in 2021/22 to 8 pp in 2022/23, and the gap between Asian and White students fell from 6 pp in 2021/22 to 3 pp in 2022/23 (while the gap across the university widened slightly). These school-level Y1 grade gaps continued respectively at 8 pp and 4 pp in 2023/24, the second PEMENTOS cohort. Although the introduction of PEMENTOS has coincided with a reduction of the grade gaps that we set out to reduce, causation is far from proven, and it is too soon to measure any effect on the degree awarding gaps of these cohorts. The programme has expanded to 165 mentees in 2024/25 and will be expanded beyond the school through APP funding [5].

## The Kingston University Academic Peer Mentoring Scheme

### Background and demographics

At Kingston University, the Academic Peer Mentoring Programme seeks to foster an inclusive learning environment by facilitating peer-to-peer support to encourage social and cultural integration between students as well as enhancing learning outcomes for the mentees. Although open and advertised to all, particular emphasis is placed on supporting BGM students and students from lower socio-economic backgrounds. The initiative responds directly to strategic institutional goals, including those related to Kingston University’s APP commitment to reducing the BGM awarding gap [13]. The APP commits the university to invest in activity that will widen participation and support the success of students from all backgrounds [13]. One strand of this endeavour has been the development of an Inclusive Curriculum Framework which sought ‘to enhance equality of opportunity all the way through the student journey by integrating multiple narratives of inclusion into a holistic framework’ [14]. The Academic Peer Mentoring Programme was one such narrative developed from 2016/17 onwards and covered all UG programmes at Kingston.

A university-wide review of the programme in 2019/20 provides the most detailed description of the scheme and its efficacy. In that academic year, the programme was embedded in 27 courses with 250 mentors recruited annually, facilitating the learning of over 2591 Level 4 and Level 5 students (13.9% of the university’s UG population). In total, 234 mentors took part in the programme (1.3% of the university UG population). Table 1 shows a demographic analysis of mentees and mentors that year; the profiles were similar in subsequent years. In line with the purposes of the programme, most of the academic mentees were from BGM backgrounds and were more likely than the average Kingston undergraduate to come from a deprived area (IMD Q1/2), to commute to campus and to have entered university with a relatively low entry tariff, with BTEC qualifications and/or via UCAS Clearing. In contrast, mentors were less likely than the average undergraduate to possess these characteristics.

**Table 1:** Comparison of demographic and educational characteristics across three groups: Kingston University’s overall UG population, mentees and mentors

Group	UG population (%)	Mentees (%)	Mentors (%)
Asian (incl. Chinese)	26.5	32.3	20.6
Black	21.6	22.7	11.0
Mixed/Other	14.0	16.8	14.0
White	36.8	27.1	52.6
Learning difficulty	6.5	4.7	5.7
Other disability	10.1	8.7	12.3
No disability	83.4	86.6	82.0
Female	57.8	43.3	68.9
Male	41.8	56.2	30.3
Mature^†^	33.1	21.1	32.9
Young	66.9	78.9	67.1
1st Gen to HE	44.8	45.8	41.2
Not 1st Gen to HE	40.0	38.1	41.2
IMD Q1, Q2	43.0	47.3	34.6
IMD Q3, Q4, Q5	50.6	46.4	53.5
Commuter	52.5	58.3	38.2
Has a BTEC	33.3	36.3	16.2
Clearing	26.5	29.9	23.7
Average tariff^*^	106.5	93.7	117.3

† Mature students were at least 21 years old when they began the course.

* Values in this row are mean UCAS entry tariff scores.

### Scheme design

Recognising disciplinary differences and local pedagogical needs, the programme adopts a flexible, department-led approach to implementation. Three primary models of delivery have been established, allowing academic departments to adapt the mentoring model in alignment with curricular requirements and student needs:

- **Mentor-led model:** Mentors conduct supplementary sessions outside of the scheduled curriculum, offering targeted assistance to mentees.
- **Embedded model:** Mentors are integrated into the academic timetable, providing support during lectures, seminars, tutorials, studio sessions and laboratories.
- **Bespoke model:** Tailored support is provided to meet the specific needs of individual modules.

**Figure 3:**
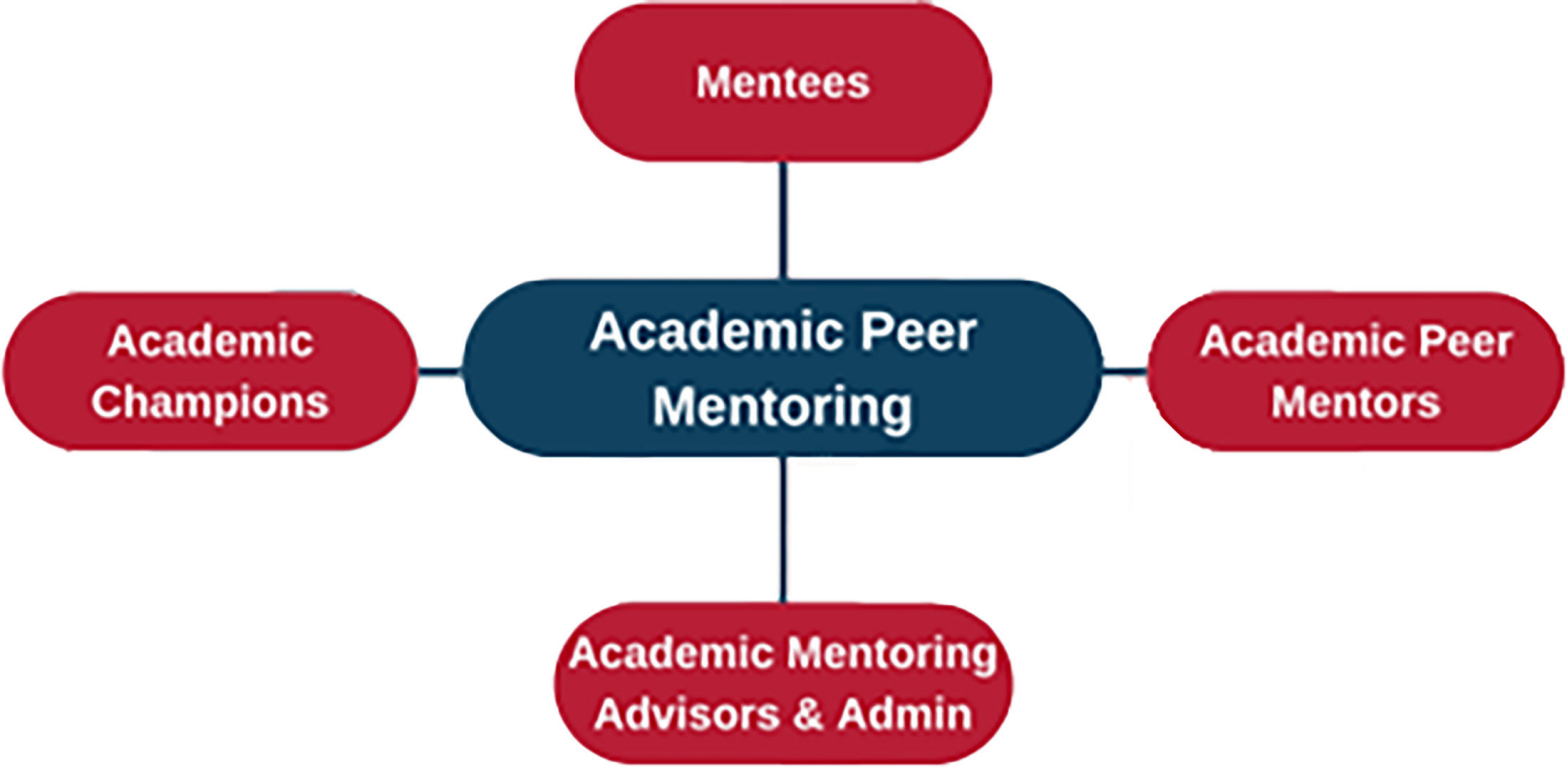
Participants in, and organisation of, the Kingston University Academic Peer Mentoring Programme.

Each department or course appoints an Academic Peer Mentoring Champion, an academic staff member who chooses the best delivery mode for their learners and is responsible for overseeing the local implementation of the mentoring scheme. These Champions collaborate closely with the Academic Mentoring Advisers, who provide ongoing support and strategic guidance (Figure 3). The Academic Mentoring Advisers are paid Professional Services Staff, whose time is almost fully assigned to the programme and who are based in the Student Development and Graduate Success section of the Directorate for Students at Kingston. Support materials and resources include the following and ensure consistency in programme delivery while allowing room for innovation:

- A comprehensive Mentors’ Toolkit
- Handbooks for both academic leads and mentors
- Impact evaluation templates and guidance
- Digital platforms to facilitate the sharing of best practices

One particularly successful version of the Academic Peer Mentoring Programme was run in the undergraduate Pharmaceutical Science course at Kingston. The model employed here was the mentor-led model where timetabled sessions were set up around assessment pinch points in an Academic Skills module. Mentors were second-year Pharmaceutical Science students paid at London Living Wage levels to mentor first year, Level 4 students. The mentors were given training which emphasised that they should not just deliver up solutions to problems but guide the first-year student mentees through problem-solving activities so that they ‘learned how to learn’. Mentors often guided mentees through topics that were found to provide particular challenges to first-year students, such as an introductory Biosciences module and organic and medicinal chemistry, in preparation for summative coursework deadlines. These assessments included intra-semester MCQ-type tests, written practical reports and data analysis exercises.

### Results and discussion

Table 2 shows demographically detailed module-level results from that year’s Pharmaceutical Science course. Mentees in virtually every identifiable student sub-population achieved better outcomes than non-mentees. Overall, the pass rates of mentees were over 22 pp greater than non-mentees in this module.

**Table 2:** Module outcomes for mentees in various demographic groups in the Level 4 Academic Skills module of the Pharmaceutical Science course in 2019/20

Group	Non-Mentees: Total FPE	Non-Mentees: % Passed	Mentees: Total FPE	Mentees: % Passed
Asian (incl. Chinese)	15	73.3	22	100.0
Black	11	72.7	12	91.7
Mixed/Other	5	100.0	5	100.0
White	9	77.8	5	100.0
Learning difficulty	0	-	5	100.0
Other disability	5	100.0	5	80.0
No disability	39	74.4	34	100.0
Female	11	72.7	30	96.7
Male	29	75.9	11	100.0
Mature	5	60.0	6	100.0
Young	35	77.1	35	97.1
1st gen to HE	24	70.8	21	95.2
Not 1st gen to HE	15	86.7	19	100.0
IMD Q1, Q2	30	73.3	28	96.4
IMD Q3, Q4, Q5	8	75.0	13	100.0
Overall	40	75.0	41	97.6

Numbers of students (total full person equivalent (Total FPE)) and pass rates are shown for members of each group who did, or did not, participate in the Academic Peer Mentoring Programme. Numbers between 1 and 5 are rounded to 5. ‘1st gen to HE’ indicates students whose parents were not educated to degree level, and mature students were at least 21 years old when they began the course.

The programme appeared particularly successful with BTEC students. Across the School of Life Sciences, Pharmacy and Chemistry (within which school lies the Pharmaceutical Science degree), it was found that mentees who entered university with a BTEC as their highest qualification were much more likely than non-mentee BTEC students to progress (80.8% compared with 59.8%, out of 156 BTEC students) and had higher progression at first attempts rates compared to non-mentee BTEC students (64.1% compared with 43.6%). A similar result was found for the 240 students commuting for 50 min or more, with 87.1% of mentee commuting students passing compared with 62.8% of non-mentee commuting students, and 72.5% of mentee commuting students passing at first attempt compared with 48.1% of non-mentee commuting students.

**Figure 4:**
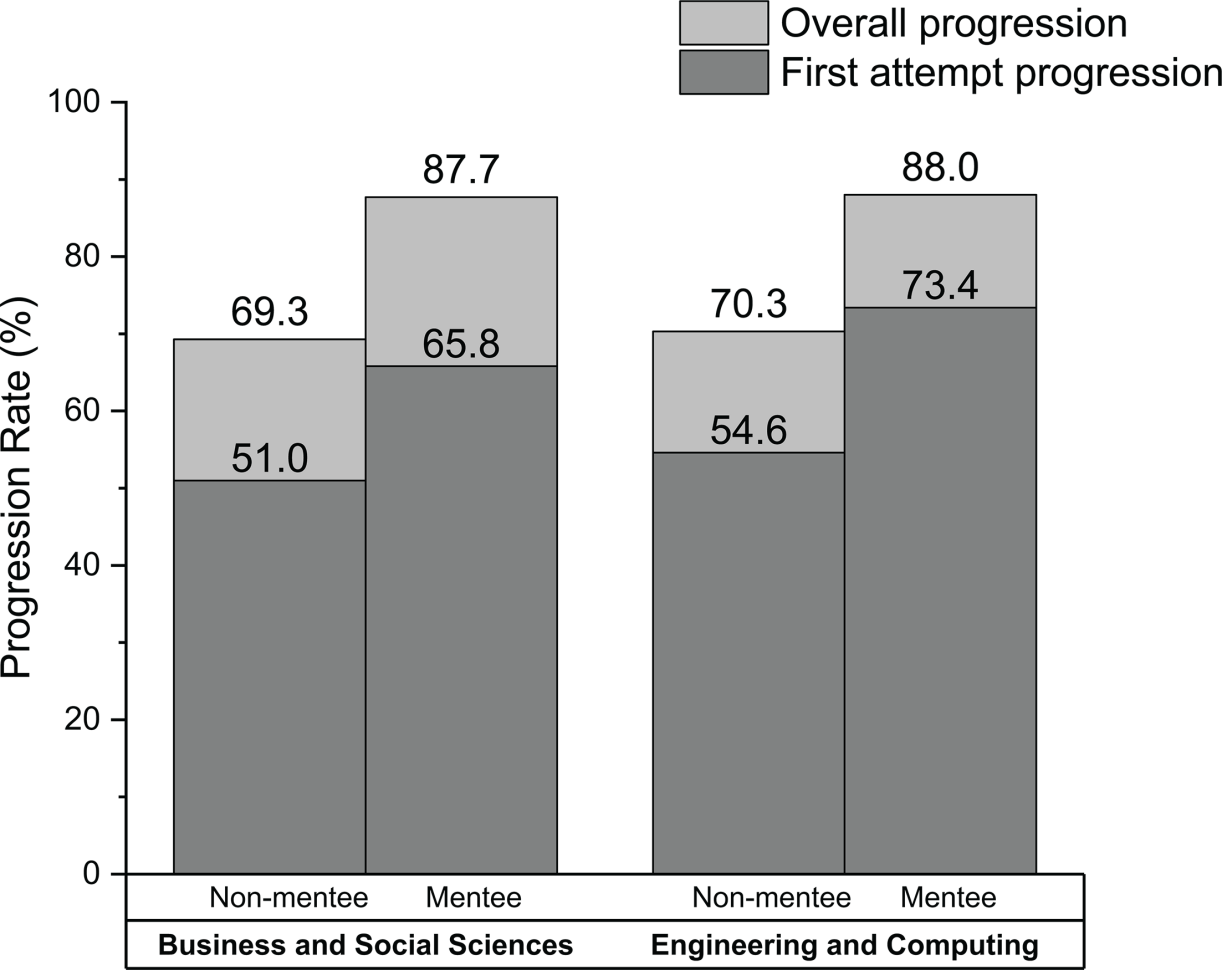
2019/20 progression rates of mentees and non-mentees in the Faculty of Business and Social Sciences and the Faculty of Engineering and Computing at Kingston University.

Progression rates from 2019/20 across two faculties are shown in Figure 4. A chi-square test of independence showed that academic mentees progressed at significantly higher rates than the average student, both at first attempt and after re-assessment. This association was significant for both faculties and for all levels of study.

**Figure 5:**
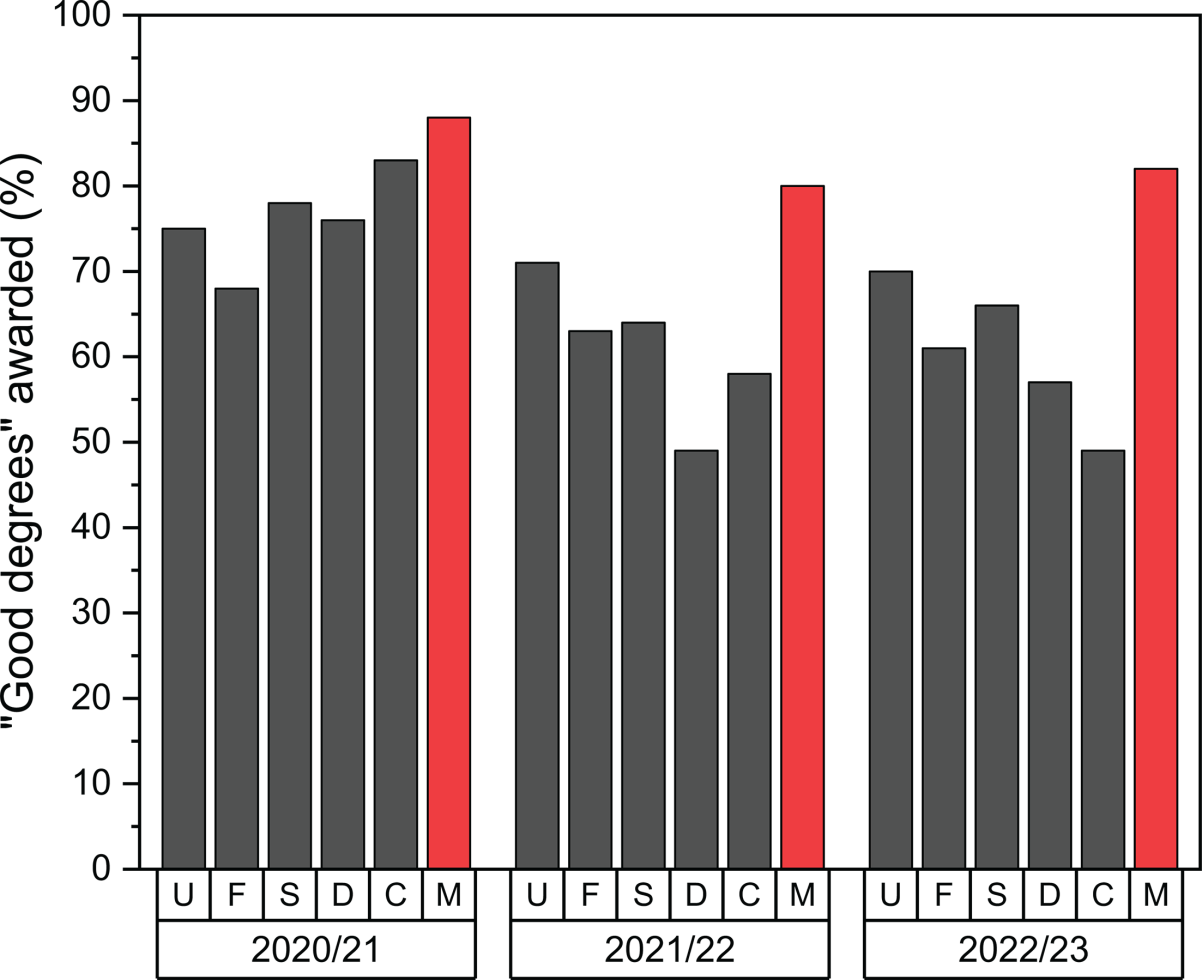
Percentages of ‘good degrees’ (1st and 2:1 classifications) awarded over three academic years at Kingston University to students within the following groups: University (**U**), Faculty of Science, Engineering and Computing (**F**), School of Life Sciences, Pharmacy and Chemistry (**S**), Department of Chemical and Pharmaceutical Sciences (**D**), Course of Pharmaceutical Science (**C**), Academic Peer Mentoring Mentees (**M**).

A longitudinal analysis of the Academic Peer Mentoring Programme was carried out in the Pharmaceutical Science degree and in the institutional levels above this course (Figure 5). The benefits of the programme were found to be maintained for three years after 2019/20 despite the wide ramifications of the COVID pandemic.

Other studies have identified similar benefits of peer mentoring programmes [15,16], including the development of greater self-awareness and confidence by mentors [17]. While the Kingston University Academic Peer Mentoring programme bears some resemblance to other schemes (elsewhere called Peer Assisted Learning (PAL), Peer Mentoring, Student Support Mentoring, Learning Development Mentoring etc. [18]) it is distinguished by a number of features: 1) the Academic Peer Mentoring sessions were embedded in the curriculum and were visible on students’ automated timetables; 2) the sessions were timetabled around major summative assessments; 3) mentors were comprehensively trained and paid for their efforts and, having progressed to the next level of their studies, provided exemplars of success to the first year mentees; 4) the programme was led by one identifiable academic in each case, designated as the Academic Peer Mentoring Champion for a course [19]. All of these features gave both implicit and explicit signals of the students' value and relevance of these sessions. Peer-assisted learning programmes, on the other hand, are often characterised by a reciprocal learning process whereby the development of knowledge, skills and competences benefits both parties more or less equally [15].

## Conclusion

The PEMENTOS scheme at Royal Holloway University of London and the Academic Peer Mentoring Programme at Kingston University demonstrate the potential of structured, inclusive peer mentoring programmes to address both transitional challenges for new students and persistent awarding gaps in UK higher education. While differing in scope and delivery modes, both programmes fostered a greater sense of belonging to a learning community, improved engagement and were associated with improved academic outcomes for underrepresented student groups. It is important to note that our results are ‘Type 2’ APP evidence – empirical studies which show that the interventions are associated with benefits but did not necessarily cause them [20]. This is because both programmes were open to all students, and therefore lacked demographically matched control groups.

In both schemes, mentors were paid for their work, which took place within a structured timetable of activity. These are key elements common to both programmes that may be important for the successful implementation of similar schemes in the life sciences. Other researchers have found that ‘protected and regular times for meeting’ are important for the success of peer mentoring relationships [21], and that paying mentors allows them to devote time to the peer mentoring relationship that they otherwise might spend on outside employment [22]. Both programmes highlight the reciprocal nature of mentoring; mentees gain confidence, academic skills and social integration from students who have recently experienced the same transition to university, while mentors benefit from meta-cognitive development, self-reflection and transferable professional skills [15-17]. Peer mentoring schemes such as those described in this work offer a scalable, flexible and evidence-informed approach to enhancing life-science student success in the post-pandemic HE landscape.

## Open Access

This article has been published open access under our Subscribe to Open programme, made possible through the support of our subscribing institutions, learn more here: https://portlandpress.com/pages/open_access_options_and_prices#conditional

## Abbreviations

APP: access and participation plan
BGM: Black and global majority
BTEC: Business and Technology Education Council
COVID: coronavirus disease
EDI: equality, diversity and inclusion
FPE: full person equivalent
HE: higher education
HUBS: Heads of University Biosciences
IMD: Index of Multiple Deprivation
MCQ: multiple-choice question
PAL: peer-assisted learning
PEMENTOS: Peer Mentoring to Succeed
POLAR4: Participation of Local Areas 4
UCAS: Universities and Colleges Admissions Service
UG: undergraduate
VLE: virtual learning environment
